# Supplementary material for: Comparing a novel machine learning method to the Friedewald formula and Martin-Hopkins equation for low-density lipoprotein estimation
Source: PLoS One. 2020 Sep 30;15(9):e0239934. doi: 10.1371/journal.pone.0239934 (PMC7526877; doi:10.1371/journal.pone.0239934)
Supplement: S1 Table — Improved accuracy of LDL-C estimation using the Weill Cornell model results in significant reclassification of LDL-C values across guideline determined LDL-C thresholds (in mg/dL) compared to the (A) Friedewald formula and (B) Martin Hopkins equation. Results are shown for the validation set. (DOCX) [file pone.0239934.s001.docx]

**S1 Table. Improved accuracy of LDL-C estimation using the Weill Cornell model results in significant reclassification of LDL-C values across guideline determined LDL-C thresholds (in mg/dL) compared to the (A) Friedewald formula and (B) Martin Hopkins equation. Results are shown for the validation set.**

| **A** | **Friedewald Formula** | | |  |  |  |  |  |  |  |  |  |
| --- | --- | --- | --- | --- | --- | --- | --- | --- | --- | --- | --- | --- |
| **Weill Cornell Model** | **0-70** | | **70-100** | | **100-130** | | **130-160** | | **160-190** | | **>190** | |
| **0-70** | **302** |  | **18** |  | **0** |  | **0** |  | **0** |  | **0** |  |
|  | *278* | 24 |  |  |  |  |  |  |  |  |  |  |
|  |  |  | 18 |  |  |  |  |  |  |  |  |  |
| **70-100** | **150** |  | **784** |  | **18** |  | **1** |  | **0** |  | **0** |  |
|  |  | 3 | 694 | 90 |  |  |  | 1 |  |  |  |  |
|  | 123 | 24 |  |  | 15 | 3 |  |  |  |  |  |  |
| **100-130** | **1** |  | **156** |  | **618** |  | **17** |  | **0** |  | **0** |  |
|  |  |  |  | 5 | *537* | 81 |  |  |  |  |  |  |
|  | 1 |  | 127 | 24 |  |  | 15 | 2 |  |  |  |  |
| **130-160** | **0** |  | **0** |  | **113** |  | **180** |  | **10** |  | **0** |  |
|  |  |  |  |  |  | 2 | *239* | 41 |  |  |  |  |
|  |  |  |  |  | 84 | 27 |  |  | 9 | 1 |  |  |
| **160-190** | **0** |  | **0** |  | **0** |  | **73** |  | **106** |  | **5** |  |
|  |  |  |  |  |  |  |  | 1 | *82* | 24 |  |  |
|  |  |  |  |  |  |  | 48 |  |  |  | 5 |  |
| **>190** | **0** |  | **0** |  | **0** |  | **0** |  | **16** |  | **40** |  |
|  |  |  |  |  |  |  |  |  |  |  | *35* | 5 |
|  |  |  |  |  |  |  |  |  | 12 | 4 |  |  |

| **B** | **Martin-Hopkins Equation** | | |  |  |  |  |  |  |  |  |  |
| --- | --- | --- | --- | --- | --- | --- | --- | --- | --- | --- | --- | --- |
| **Weill Cornell Model** | **0-70** | | **70-100** | | **100-130** | | **130-160** | | **160-190** | | **>190** | |
| **0-70** | **305** |  | **15** |  | **0** |  | **0** |  | **0** |  | **0** |  |
|  | *281* | 24 |  |  |  |  |  |  |  |  |  |  |
|  |  |  | 15 |  |  |  |  |  |  |  |  |  |
| **70-100** | **89** |  | **839** |  | **24** |  | **1** |  | **0** |  | **0** |  |
|  |  |  | 752 | 87 |  | 1 |  | 1 |  |  |  |  |
|  | 59 | 30 |  |  | 21 | 2 |  |  |  |  |  |  |
| **100-130** | **0** |  | **104** |  | **673** |  | **15** |  | **0** |  | **0** |  |
|  |  |  |  |  | *576* | 97 |  |  |  |  |  |  |
|  |  |  | 89 | 15 |  |  | 15 |  |  |  |  |  |
| **130-160** | **0** |  | **0** |  | **100** |  | **295** |  | **8** |  | **0** |  |
|  |  |  |  |  |  | 1 | *256* | 39 |  |  |  |  |
|  |  |  |  |  | 70 | 29 |  |  | 6 |  |  |  |
| **160-190** | **0** |  | **0** |  | **0** |  | **68** |  | **110** |  | **6** |  |
|  |  |  |  |  |  |  |  |  | *85* | 25 |  |  |
|  |  |  |  |  |  |  | 44 | 24 |  |  | 6 |  |
| **>190** | **0** |  | **0** |  | **0** |  | **0** |  | **17** |  | **39** |  |
|  |  |  |  |  |  |  |  |  |  |  | *34* | 5 |
|  |  |  |  |  |  |  |  |  | 13 | 4 |  |  |

**Bold**: total.

*Italic*: all accurate.

Blue: all inaccurate.

Green: Weill Cornell model accurate while other models inaccurate.

Red: Weill Cornell model inaccurate while other models accurate.
